# Learning models for forecasting hospital resource utilization for COVID-19 patients in Canada

**DOI:** 10.1038/s41598-022-12491-z

**Published:** 2022-05-24

**Authors:** Jianfei Zhang, Harini Sanjay Pathak, Anne Snowdon, Russell Greiner

**Affiliations:** 1 Department of Computing Science, University of Alberta, Edmonton, AB Canada; 2 Alberta Machine Intelligence Institute (Amii), Edmonton, AB Canada; 3 Odette School of Business, University of Windsor, Windsor, ON Canada; 4 Supply Chain Advancement Network in Health (SCAN Health), Windsor, ON Canada; 5 Département d’informatique, Université de Sherbrooke, Sherbrooke, QC Canada

**Keywords:** Computer science, Epidemiology, Health policy, Machine learning, Data mining

## Abstract

Hospitals in Canada are facing a crisis-level shortage of critical supplies and equipment during the COVID-19 pandemic. This motivates us to create predictive models that can use Canada COVID-19 data and pandemic-related factors to accurately forecast 5 quantities—three related to hospital resource utilization (i.e., the number of hospital beds, ICU beds, and ventilators that will be needed by COVID-19 patients) and two to the pandemic progress (i.e., the number of COVID-19 cases and COVID-19 deaths)—several weeks in advance. We developed a machine learning method that can use information (i.e., resource utilization, pandemic progress, population mobility, weather condition, and public policy) currently known about a region since March 2020, to learn multiple temporal convolutional network (TCN) models every week; each used for forecasting the weekly average of one of these 5 quantities in Canada (respectively, in six specific provinces) for each, in the next 1 (resp., 2,3,4) weeks. To validate the effectiveness of our method, we compared our method, versus other standard models, on the COVID-19 data and hospital resource data, on the tasks of predicting the 116 values (for Canada and its six most populated provinces), every week from Oct 2020 to July 2021, and the 20 values (only for Canada) for four specific times within 9 July to 31 Dec 2021. Experimental results show that our 4640 TCN models (each forecasting a regional target for a specific future time, on a specific date) can produce accurate 1,2,3,4-week forecasts of the utilization of every hospital resource and pandemic progress for each week from 2 Oct 2020 to 2 July 2021, as well as 80 TCN models for each of the four specified times within 9 July and 31 Dec 2021. Compared to other baseline and state-of-the-art predictive models, our TCN models yielded the best forecasts, with the lowest mean absolute percentage error (MAPE). Additional experiments, on the IHME COVID-19 data, demonstrate the effectiveness of our TCN models, in comparison with IHME forecasts. Each of our TCN models used a pre-defined set of features; we experimentally validate the effectiveness of these features by showing that these models perform better than other models that instead used other features. Overall, these experimental results demonstrate that our method can accurately forecast hospital resource utilization and pandemic progress for Canada and for each of the six provinces.

## Introduction

As of March 2022, COVID-19 has infected more than 3.4 million and killed more than 37 thousand people in Canada^1^. The rapid increases in patient volumes during waves of this pandemic overwhelmed health systems in many regions (e.g., Ontario). There is now an urgency for creating predictive tools to accurately predict the utilization of hospital resources (e.g., ICU beds), in a timely manner (e.g., 4 weeks in advance), so that health system leaders and decision-makers can accurately prepare for these critical supplies/equipment necessary to care for patients infected with the virus. Many projects have attempted to forecast regional resources^2^—e.g., the hospital beds^3^ and the ICU beds^4^ that will be needed for COVID-19 patients in Ontario, in 4 weeks. Different from those previous projects, we make multiple forecasts that involve five targets, seven regions, and four time horizons (summarized in Table 1). The five forecasting targets include the three quantities in terms of hospital resources required to accommodate COVID-19 patients in Canada (i.e., the number of hospital beds, ICU beds, and ventilators), and the two quantities in terms of pandemic progress highly relevant to the hospital resource utilization (i.e., the number of COVID-19 cases and COVID-19 deaths). Due to the uneven spread of the disease across the Canadian provinces, it is useful to accurately forecast hospital resource utilization not only for all of Canada, but also for each province individually—here, we forecast each target for Canada and for the six most populated provinces: Alberta (AB), British Columbia (BC), Manitoba (MB), Ontario (ON), Québec (QC), and Saskatchewan (SK), for each of the next 4 weeks—i.e., for 1-week (day 1 [“*tomorrow*”]–day 7), 2-week (day 8–day 14), 3-week (day 15–day 21), and 4-week (day 22–day 28).

**Table 1:** The 5 targets, 7 regions, and 4 horizons of our forecasting models.

Target	Region (abbreviation)	Horizon (days in the future)
Hospital beds ICU beds Ventilators Cases Deaths	Canada (CA) Alberta (AB) British Columbia (BC) Manitoba (MB) Ontario (ON) Québec (QC) Saskatchewan (SK)	1-week (day 1–day 7) 2-week (day 8–day 14) 3-week (day 15–day 21) 4-week (day 22–day 28)

**Figure 1:**
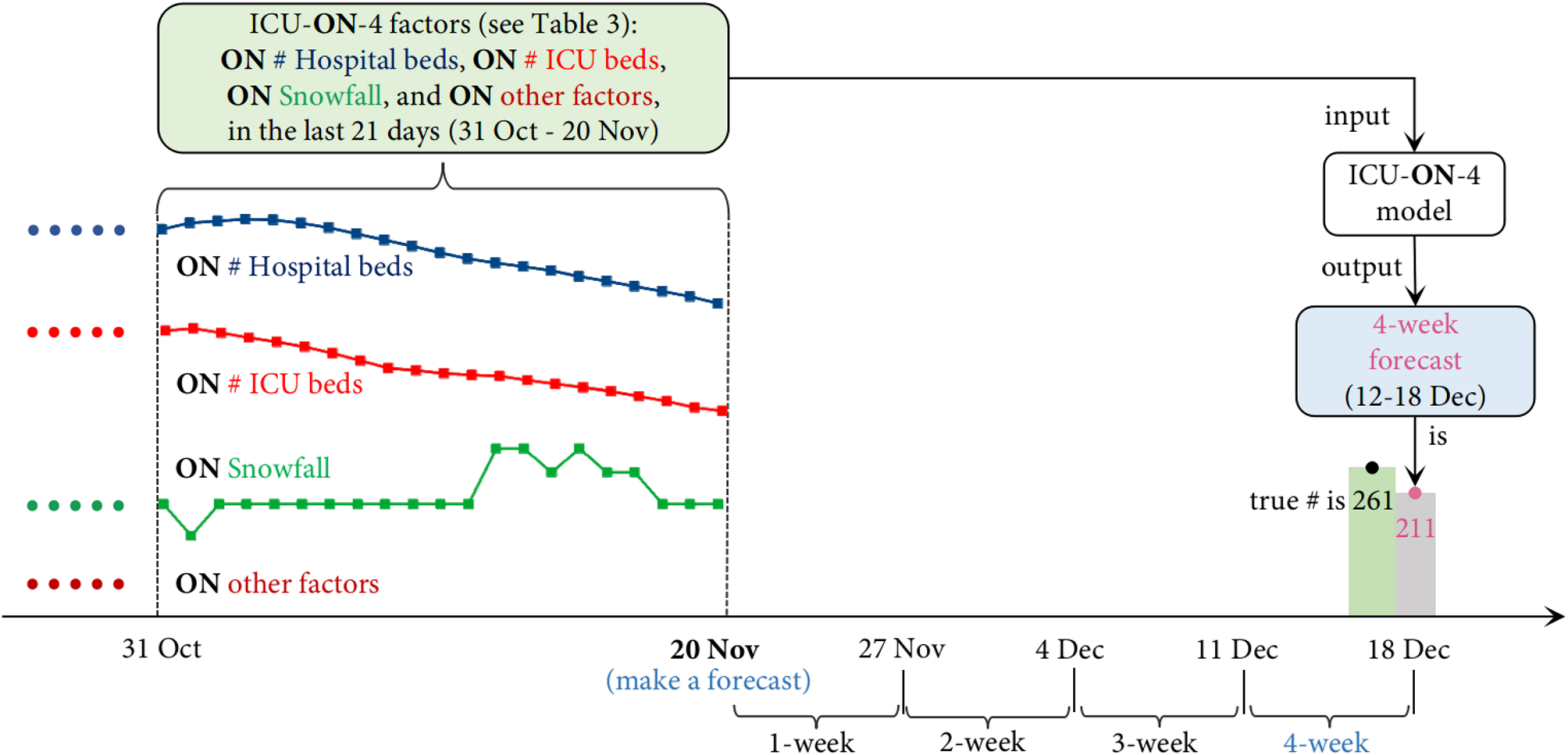
An example of target-region-horizon-specific forecast: forecasting the weekly average number of ICU beds (*target*) needed by COVID-19 patients in Ontario (*region*) for 4-week (*horizon*)—i.e., for 22 to 28 days after 20 Nov 2020 (that is, 12–18 / Dec).

**Figure 2:**
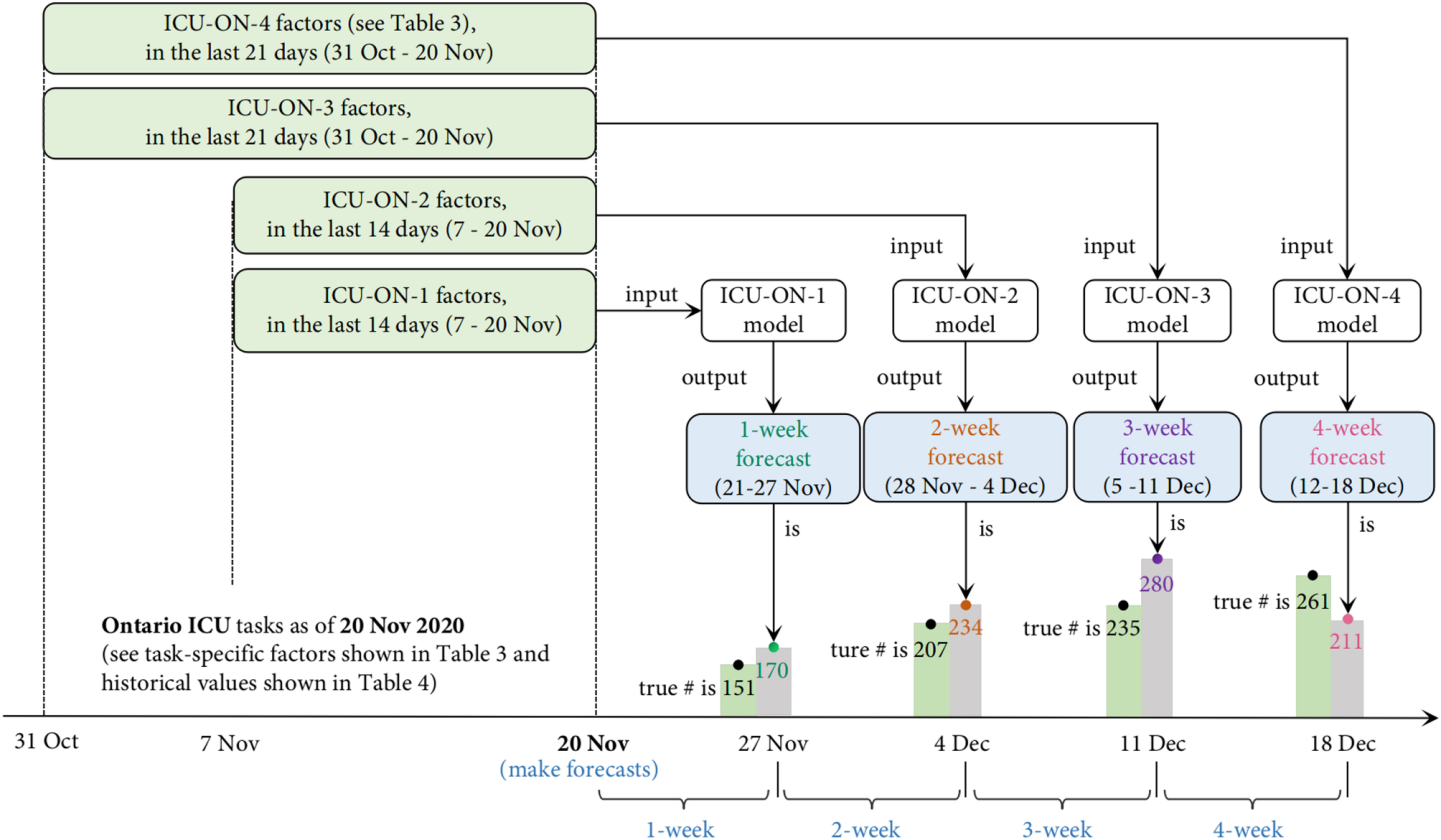
ICU-ON-1, ICU-ON-2, ICU-ON-3, and ICU-ON-4 forecasts, each made on 20 Nov 2020.

Forecasting the pandemic progress and hospital resource utilization is challenging as the spread of the disease has been highly variable over both time and region. Hence, every week, we created various forecasting models for each regional target for each time horizon (from a Saturday to a Friday). For example, on Friday 20 Nov 2020, we built an ICU-ON-4 model which predicted that Ontario (region) may need 211 ICU beds (target) for 4-week (i.e., the week of 12–18 Dec); this forecast, as shown in Fig. 1, is close to the truth: on weekly average (i.e., the average daily number over a 7-day period), 261 ICU beds were actually occupied in Ontario by COVID-19 patients for that week. For the 5 targets, 7 regions, and 4 horizons, we have 116 forecasting tasks (4 horizons × [6 provinces × 4 tasks/province + 5 tasks for Canada]) every week between 2 Oct 2020 and 2 July 2021, and 20 tasks for each of four specific times between 9 July and 31 Dec 2021. On a weekly basis (i.e., every Friday), we build 116 different models (between 2 Oct 2020 and 2 July 2021) and 20 models (between 9 July and 31 Dec 2021), each for a target-region-horizon-specific forecast—e.g., the ICU-ON-4 model (learned on 20 Nov 2020) is for the 4-week forecasted number of ICU beds in Ontario, for the week ending 18 Dec 2020. For this location and date, we also learned and used three other models (i.e., ICU-ON-1 model, ICU-ON-2 model, ICU-ON-3 model) to make ICU-ON-1,-2,-3 forecasts (for the weeks ending 27 Nov, 4 Dec, 11 Dec, respectively); see Fig. 2.

Towards learning each such forecasting model, we collect known data (from 21 March 2020 until the date when the prediction is made) every week about the daily statistics on the 27 factors, including 3 resource utilization factors, 2 pandemic progress factors, 5 population mobility factors, 5 weather condition factors, and 12 public policy factors. In addition to factors corresponding to what we are predicting (the “target” values), we collect other factors—here about mobility, weather, and policy—as they have effects on the average contact rates and therefore on the pandemic progress and resource utilization. Each target-region-horizon-specific model uses a different subset of the features—each a different specific subset of the 27 factors (rather than on all the 27 factors) over a specific number of previous weeks. For example, the ICU-ON-4 (20 Nov 2020) model shown in Fig. 1 uses the number of hospital beds and ICU beds, snowfall, and the value of other factors over the 21 days prior to 20 Nov 2020 (i.e., 31 Oct–20 Nov), whereas the ICU-ON-1, ICU-ON-2, ICU-ON-3 models use different subsets of factors; Table 3 presents the actual factors used for such forecasts (see discussion below).

Essentially, each task is a multivariate time-series forecasting problem^5^—i.e., utilizing those COVID-19-related factors to predict a single future target value (i.e., the weekly average value of a specific week, ending on Friday). Given that *temporal convolutional network* (TCN)^6^ model has worked successfully in many multivariate time-series modeling and forecasting tasks—e.g., stock prediction^7^, energy forecasting^8^, and traffic flow forecasting^9^—we decided to build TCN models for our forecasting tasks. TCNs can leverage their special convolutional network architecture^6^ to easily look back the past values of the time series (e.g., the number of hospital beds and ICU beds in the past 21 days) and explore the relationship between past and future values, based on which we can accurately forecast the future target value given the past values.

For each of the 40 weeks between 2 Oct 2020 and 2 July 2021, we conduct a total of 4640 (40 weeks × 116 tasks/week) experiments. Our experimental results (“Results” section) demonstrate that our method can produce more accurate forecasts of hospital resource utilization, i.e., with mean absolute percentage error (MAPE) that is lower than the four baseline and state-of-the-art methods—i.e., SEIR (such as PHAC-SEIR^10^ and DELPHI-SEIR^11^), ARIMA^12^, XGBoost^3^, and LSTM^13^. We also conduct 80 (4 times × 20 tasks/time for Canada) experiments, using TCN and PHAC-SEIR to forecast the five targets in the whole of Canada at four time points (i.e., 30 July, 10 Sept, 22 Oct, 3 Dec 2021) during the fourth pandemic wave. The experimental results reveal that the TCN models can more accurately forecast each target for the next 4 weeks, achieving lower MAPEs, lower than PHAC-SEIR—i.e., the standard SEIR model used by the Public Health Agency of Canada (PHAC).

Each of our TCN models used a specific pre-defined set of factors (see Table 2); to investigate the effectiveness of using these task-specific factors, we compared those models, to other TCN models that used various other factors and histories; the results show that our current task-specific factors are more effective than those other options. As a final set of experiments, we compared our forecasts to the results of the IHME project^14^, which provides forecasts of the number of hospital beds, ICU beds, cases, and deaths, based on different COVID-19 and hospital resource data. Here, we found that our TCN approach performed better, compared to the IHME’ forecasts—which reinforces our claim that our method can make accurate forecasts based on different COVID-19 ground truth data (Note there have been various ground-truth data provided by Johns Hopkins University^15^, New York Times^16^, University of Oxford^17^, etc.), not only based on the current ArcGIS^18^ data.

## Data

Every Friday (when we make the forecast—e.g., 20 Nov in Fig. 2), we collect and update the data in terms of 27 factors (shown in Table 2). Each factor has a numeric value each day from 21 March 2020 to the current date. Here, resource utilization and pandemic progress are also our ground-truth for evaluating the models’ performance.

- *Resource utilization*: The daily number of hospital beds and ICU beds occupied by COVID-19 patients in Canada and in each province are provided by ArcGIS^18^. The daily number of ventilators is only available for Canada released by the Public Health Agency of Canada^1^; the provincial number of ventilators is not available.
- *Pandemic progress*: The daily number of new COVID-19 cases, and new COVID-19 deaths, in Canada and in each province, is provided by ArcGIS^18^.
- *Population mobility*: The mobility data, available from the community mobility reports^19^, provide insights into the daily community movement trends over time by geography, across different categories of places, such as retail and recreation, groceries and pharmacies, parks, transit stations, and residential.
- *Weather condition*: The weather data from NOAA^20^ include daily meteorological information about Canada and provinces: average temperature, rainfall, relative humidity, dew point, and snowfall. (Note the only field with missing values is for snowfall; we fill in the missing values via the *k*-nearest-neighbor imputation^21^ with *k* = 3).
- *Public policy*: The policy data provided by the University of Oxford^17^ are measures of government daily responses to COVID-19, which are recorded on ordinal or continuous scales for 12 policy areas; see Table 2.

**Table 2:** The 27 factors, sorted into resource utilization, pandemic progress, population mobility, weather condition, and public policy.

Category	Total # of factors	Factors	Type of daily single value
Resource utilization	3	Hospital beds	Non-negative integer
		ICU beds	Non-negative integer
		Ventilators	Non-negative integer
Pandemic progress	2	Cases	Non-negative integer
		Deaths	Non-negative integer
Population mobility	5	Retail and recreation	Integer
		Groceries and pharmacies	Integer
		Parks	Integer
		Transit stations	Integer
		Residential	Integer
Weather condition	5	Average temperature	Real (celsius)
		Rainfall	Non-negative real (mm)
		Relative humidity	Non-negative real (%)
		Dew point	Real (celsius)
		Snowfall	Non-negative real (mm)
Public policy	12	School closing	{0,1,2,3}
		Workplace closing	{0,1,2,3}
		Cancel public events	{0,1,2}
		Restrictions on gatherings	{0,1,2,3,4}
		Public transport closing	{0,1,2}
		Stay at home	{0,1,2,3}
		Restrictions on internal movement	{0,1,2}
		International travel	{0,1,2,3,4}
		Public information campaigns	{0,1,2}
		Testing policy	{0,1,2,3}
		Contact tracing	{0,1,2}
		Facial coverings	{0,1,2,3,4}

The numeric value for each factor, for each time and each region, is the average of the 7 daily values, associated with the 7th day.

## Methodology

### Model’s input

Every week, we learn 116 different TCN models, each for a target-region-horizon-specific forecasting task between 2 Oct 2020 and 2 July 2021. During the fourth pandemic wave, we learned 20 different TCN models on 30 July, 10 Sept, 22 Oct, and 3 Dec 2021, respectively. Each TCN model we learned for a target-region-horizon-specific task uses as input a specific subset of the factors (rather than all the 27 factors)—i.e., specific factors and their specific past values—e.g., we use the *currently known* number of deaths in the current week when estimating the *future* number of deaths. We selected these specific *task-specific* factors based on our prior knowledge and assumption about these factors:

- We consider that the change in mobility, weather, and policy will not affect pandemic progress and hospital resource utilization in the near *future* (i.e., 1 week later)—e.g., adjusting the policy about *international travel restrictions* may not alter the trend of resource utilization for the next 1 week. Hence, except for the 1-week forecasts, we use these factors for all the 2-, 3-, and 4-week forecasts (see $\dagger$ in Table 3; note this excludes the Death forecasts).
- For a time series for a single factor (like number of ICU beds), the current values are generally highly correlated to the future values. Hence, for each target (e.g., number of ICU beds), we use the value for the current time target values (e.g., currently known number of ICU beds) to forecast its future values, see $\checkmark$ in Table 3.
- The five target factors are also highly correlated with each other. Hence, we make the following assumptions:
  - The utilization of hospital beds in the future 1, 2, 3, and 4 weeks is generally affected by the current number of cases—e.g., a region that has a large number of cases will typically have more hospitalized in the future. Hence, for all the 1-,2-,3-,4-week hospital-beds forecasts, we use the number of cases as an input factor (see $\vee$ in Table 3).
  - Similarly, the future utilization of ICU beds and ventilators may be highly affected by the current hospitalizations. For this, we use the number of hospital beds as an input factor for all ICU-beds and ventilators forecasting tasks (see $\ddagger$ in Table 3). Further, we consider that the utilization in 1 week may also be affected by the number of deaths (see • in Table 3) because the ICU beds (resp., and ventilators) would be available soon after the death of patients in ICU (resp., or on ventilators).
  - As ICU patients are usually at high risk of death, we assume the death occurs within 2 weeks after entering ICU. Therefore, we use the number of occupied ICU beds for forecasting the number of deaths in 1 or 2 weeks (see $\bigstar$ in Table 3), but instead, use the number of cases for 3-week and 4-week forecasts (see $\odot$ in Table 3), as we assume that a few of the ICU-patients who were infected, will die 3 or 4 weeks later. (*N.b.*, these assumptions are made mainly for the first three waves in Canada when the 2-dose vaccination rate is low).

As shown in Table 4, to make a 1-week (or 2-week) forecast for each regional target, the learned 1-week (or 2-week) TCN model uses the values in the past 14 days of the prediction time; while to make a 3-week (or 4-week) forecast for each regional target, the learned 3-week (or 4-week) TCN model uses the values in the past 21 days of the prediction time. For example, Fig. 2 (which extends Fig. 1) presents the ICU-ON-1,-2,-3,-4 forecasts made by the four TCN models based on the data known on 20 Nov 2020. Each of these four different models uses the values of its own factors at the current time (shown in Table 3) and specific past values (shown in Table 4) to forecast the number of ICU beds that will be needed in Ontario. That is, the trained ICU-ON-1(20 Nov) model uses the values of the ICU-ON-1-specific factors in the past 14 days (7–20 Nov) and predicts the number of ICU beds to be 170 for 1-week (21–27 Nov); the trained ICU-ON-2(20 Nov) model uses the values of the ICU-ON-2-specific factors in the past 14 days (7–20 Nov) to predict it to be 234 for 2-week (28 Nov–4 Dec); the trained ICU-ON-3 (20 Nov) uses the values of the ICU-ON-3-specific factors in the past 21 days (31 Oct–20 Nov) to predict it to be 280 for 3-week (5–11 Dec); the trained ICU-ON-4(20 Nov) uses ICU-ON-4-specific factors in the past 21 days (31 Oct–20 Nov) to predict it to be 230 for 4-week (12–18 Dec). The true numbers of ICU beds for these 4 weeks are 151 (21–27 Nov), 207 (28 Nov–4 Dec), 235 (5–11 Dec), and 261 (12–18 Dec), respectively.

**Table 3:** The factors used for various target-region-horizon-specific forecasting tasks.

Forecasting task			Factors used for forecasting task							
Target (# of)	Region (7 regions)	Horizon (week)	Resource			Pandemic		Mobility	Weather	Policy
			Hosp.	ICU	Vent.	Cases	Deaths	5 factors	5 factors	12 factors
Hosp.	For each	1	$\checkmark$			$\vee$				
		2, 3, 4	$\checkmark$			$\vee$		$\dagger$	$\dagger$	$\dagger$
ICU	For each	1	$\ddagger$	$\checkmark$			•			
		2, 3, 4	$\ddagger$	$\checkmark$				$\dagger$	$\dagger$	$\dagger$
Vent.	For each	1	$\ddagger$		$\checkmark$		•			
		2, 3, 4	$\ddagger$		$\checkmark$			$\dagger$	$\dagger$	$\dagger$
Cases	For each	1				$\checkmark$				
		2, 3, 4				$\checkmark$		$\dagger$	$\dagger$	$\dagger$
Deaths	For each	1, 2		$\bigstar$			$\checkmark$			
		3, 4				$\odot$	$\checkmark$			

For each cell with a symbol, the factor (in this cell’s corresponding column) is used for forecasting the target (in this cell’s corresponding row). The same symbols indicate that the forecasts are made under the same model assumption; see Model’s Input discussion in “Methodology” section.

**Table 4:** The values of task-specific factors used as input for different forecasting tasks.

Target	Region	Horizon (week)	# of past values of the task-specific factors
For each	For each	1, 2	Values in the past 14 days
		3, 4	Values in the past 21 days

### Model training

Every week, we divide the available data (i.e., information about previous weeks) into a training set and a disjoint validation set, which are used to learn and optimize a TCN model. We learn a model on the training set, i.e., using the (*input,output*) pairs (see the example below) to estimate the model parameters (i.e., the weights of a neural network). In general, this learning process involves finding appropriate values for a set of hyper-parameters, such as the network depth and kernel size (which will be discussed later). We seek the hyper-parameters that produce the model that can make accurate forecasts for the validation set. The validation mimics the out-of-sample forecasting scenario; using a set that is disjoint from the training set reduces the chance that the optimized model will overfit^22^ when it makes a forecast given the new input. We then fix the model hyper-parameters and then learn the appropriate parameters by training over both the training and validation data sets.

In more detail: Motivated by our recently-developed COVID-19 forecast method—LaPoFaPo^23^—we use the “most recent” 10% of the training instances as validation data—e.g., in the example shown in Fig. 1, of the data known as of 20 Nov 2020, we use the (*input,output*) pairs whose outputs correspond to the dates before 23 Oct 2020 as training data and the pairs whose outputs correspond to the 28 days between 23 Oct and 20 Nov 2020 as the validation data. We implement the TCN in TensorFlow^24^, with a 0.01 learning rate and the sigmoid activation^25^. Each TCN model is trained for 500 epochs by using the Adam optimizer^26^. We employ the Bayesian optimizer^27^ to choose a value from {2, 3, 4, 5, 6, 7} for kernel size (i.e., how many different kernel weights used in the TCN), a value from {2, 4, 8, 16} for dilation rate, and a dropout rate in the range [0.01, 0.1], so that the TCN model can lead to the lowest loss on the validation data.

### Example of input and output

In the learning process we need to first transform the given time series into supervised data—i.e., a set of (*input,output*) pairs that the TCN supervised training algorithm can use to produce a model, where the input is the past values of the target-region-horizon-specific factors. To train the ICU-ON-4(20 Nov 2020) model shown in Fig. 1, we use the (*input,output*) pairs as of Friday 20 Nov 2020 as training instances; for each (*input,output*) pair, the time lag between *input* and *output* is 4 weeks. Here, the last training *output* is the number of ICU beds for 20 Nov 2020 (which is 128) and the corresponding training *input* is a linearized version of the matrix (shown in Equation 1) composed of 24 factors (i.e., the number of hospital beds and ICU beds, snowfall, facial coverings, etc.) for each of the 21 days from 3 Oct to 23 Oct 2020—hence, the *input* is a $504=24\times 21$ element vector (i.e., 24 factors × 21 values/factor).1

This $(\textit{input}, \textit{output})=([145,153,\ldots ,265, 34,36,\ldots ,73, 10.16,\ldots ,16.93, \ldots , 1.0,\ldots ,1.0], 128)$ pair is a single labeled training instance; other ICU-ON-4 labeled training instances correspond to other dates—e.g., there is another pair whose input is a 504-tuple and output is 123, for Ontario ICU beds for 19 Nov, and so forth, for 196 dates (i.e., every day from 8 May 2020 through 20 Nov 2020) (*N.b.*: While we only make forecasts for Friday, our training data corresponds to forecasts made on all days of the week). Together, these pairs form the training set used for training the ICU-ON-4(20 Nov) model. We then learn a TCN model from this training data (which involves 196 instance pairs, where the input of each pair is 504-dimensional). Note this specific learned TCN model will only make the ICU-ON-4 forecast for 18 Dec, given the input—the element vector in terms of the same task-specific factors for 21 days from 31 Oct to 20 Nov 2020 (see Fig. 1). (*N.b.*: this ICU-ON-4 (20 Nov) model differs from the other ICU-ON-4 models built to make predictions for different times—e.g., it is different from ICU-ON-4 (27 Nov), etc.). Here, we use a sliding window method^28^—e.g., for ICU-ON-4 forecast, we use a 21-day input window and a 1-day output window (which is 4 weeks apart from the input window) to slice each time series: the two windows move forward simultaneously over the ICU-ON-4 factors and generate the (*input,output*) pairs.

### Comparisons for model evaluation

To understand the effectiveness and reliability of the TCN models, we compare the MAPEs yielded by TCN models, to the MAPEs by the four baseline models—i.e., SEIR^10^, ARIMA^12^, XGBoost^3^, and LSTM^13^—which are trained every week based on our task-specific factors (shown in Table 3) for the forecast.

- The “susceptible exposed infectious removed” (SEIR) compartmental model, which is an epidemiological model that is often used to describe the spread of a disease^10,29–31^. We build two SEIR models, i.e., PHAC-SEIR^10^ proposed by the Public Health Agency of Canada (PHAC) and DELPHI-SEIR^11^ by the Delphi group at Carnegie Mellon University.
- The “autoregressive integrated moving average” (ARIMA) model, which can capture a suite of different standard temporal structures in COVID-19 time series data^12^. We set the number of autoregressive terms, nonseasonal differences, and lagged forecast errors for ARIMA as 4, 0, and 1, respectively.
- The “extreme gradient boosting” (XGBoost) method, which can effectively identify patterns in temporal data and often provides fairly accurate COVID-19 predictions^3^. For XGBoost, we set maximum depth to 2, learning rate to 0.2, tree estimators to 150, observation fraction and column fraction to 0.9.
- The “long short-term memory” (LSTM) neural network, which is an obvious candidate for analyzing COVID-19 time series due to its notable success in sequence modeling problems^13^. We train the LSTM model with two hidden layers, each including 20 fully-connected neurons, for 500 epochs via the Adam optimizer^26^ with a 0.1 learning rate. LSTM uses the past values shown in Table 4.

Additionally, we use IHME data about the number of hospital beds, ICU beds, cases, and deaths in Canada as ground truth and apply our TCN models to such data. We then compare TCN’s forecasts with IHME’s forecasts. Additionally, we compare the performance of TCN and PHAC-SEIR when forecasting the fourth pandemic wave.

### Evaluation metric

We compute the mean absolute percent error (MAPE)^32^ of the forecasts with respect to each task (note smaller MAPE values are better). Letting $T_i$ and $F_i^H$ (*H* indicates the forecasting horizon—here, $H\in \{1,2,3,4\}$ for 1-,2-,3-,4-week) be respectively the true and the forecast target (e.g., number of ICU beds) for the $i^\text {th}$ ($i\in \{1,\ldots ,N\}$) week, then the MAPE of total *N* *H*-week forecasts (each predicted *H* week(s) in advance) is

2 $$\begin{aligned} \text {MAPE}(N\ \text {target-region-horizon-specific forecasts})= &\, {} \text {MAPE}\Big (\big (F_1^H,T_1\big ), \ldots , \big (F_{N}^H, T_{N}\big )\Big )\nonumber \\= & {} \ \frac{1}{N}\sum ^{N}_{i=1}\Bigg |\frac{F_i^H-T_i}{T_i}\Bigg |\times 100. \end{aligned}$$

## Results

### TCN’s forecasts and corresponding MAPEs

**Figure 3:**
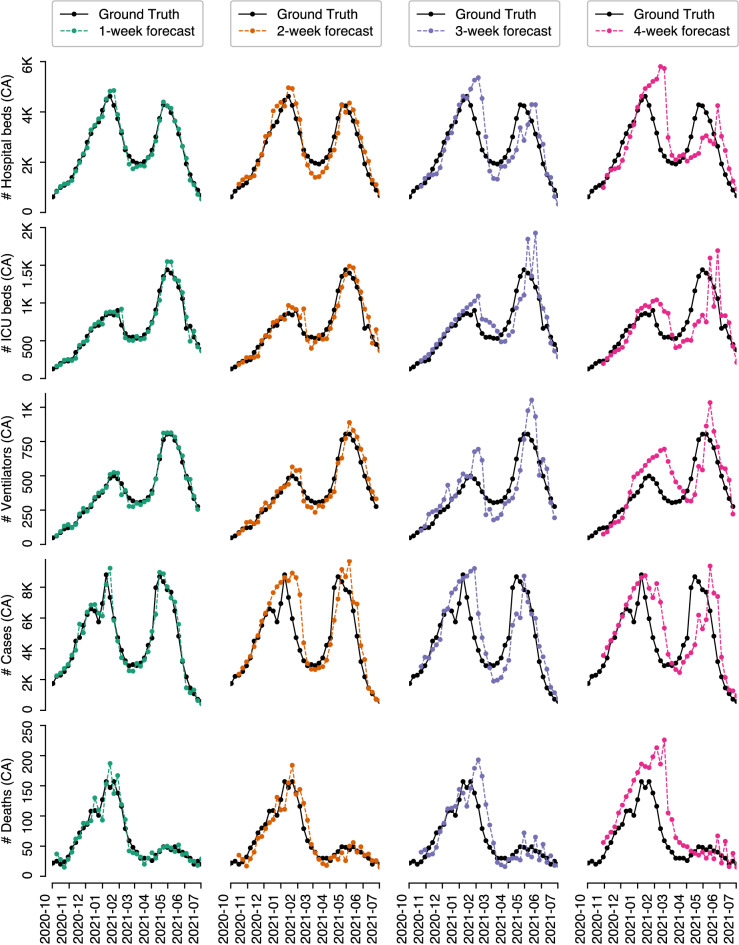
TCN’s 1-,2-,3-,4-week forecasts of the weekly average number of hospital beds, ICU beds, ventilators, cases, and deaths in *Canada* between 2 Oct 2020 and 2 July 2021.

Figure 3 shows the true (solid lines) and forecasted (dotted lines) targets (i.e., number of hospital beds, ICU beds, ventilators, cases, and deaths) for the 40 weeks between 2 Oct 2020 and 2 July 2021 in Canada, the four sub-figures in each row present the forecasts of the four targets 1, 2, 3, 4 weeks in advance, respectively. The true and forecasted number of hospital beds, ICU beds, cases, and deaths for each of the six provinces are shown in Supplemental Figs. S1–S6.

**Figure 4:**
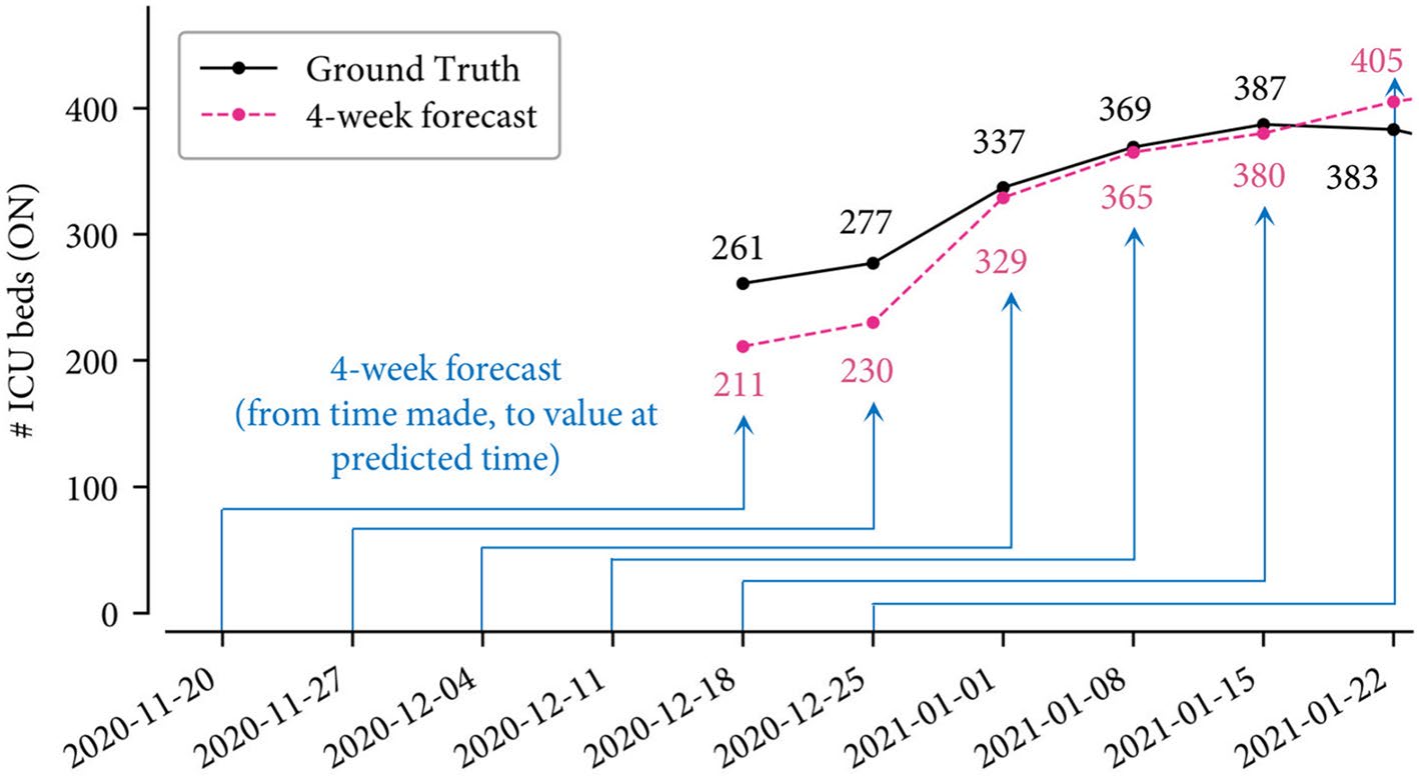
The true and ICU-ON-4 forecast number of ICU beds between 18 Dec 2020 and 22 Jan 2021.

Figure 4 shows the six ICU-ON-4 forecasts, each made 4 weeks ahead, e.g., the forecast of ICU beds in Ontario for 22 Jan 2021 is 405, which is made on 25 Dec 2020. Including such 6 forecasts, the MAPE of the total 40 ICU-ON-4 forecasts is (see Table 5)

$$\begin{aligned}&\text {MAPE}\Big (\ldots , (211,261), \ldots , (405, 383), \ldots \Big )\\&\quad = \frac{1}{40}\left( \cdots + \frac{|211-261|}{261}+\cdots +\frac{|405-383|}{383} + \cdots \right) \\&\quad = 28.76\%. \end{aligned}$$

Similarly, we compute all MAPEs of the 40 weekly forecasts with respect to each task and present the results in Table 5 (which indicates the differences between the solid line and dotted lines in each sub-figure of Fig. 3). We see that our TCN models produce accurate 1-week forecasts (with as low as 6%, 8.38%, and 9.47% MAPE of the , in Canada) but good 4-week forecasts (with as low as 32.42%, 27.26%, 33.26% MAPE for the 4-week forecast of the utilization of hospital beds, ICU beds, and ventilators, respectively, in Canada). The 4-week forecast of the number of cases and deaths in Canada leads to a 37.72% MAPE and a 56.53% MAPE, respectively.

**Table 5:** MAPEs (%) of TCN’s forecasts during the 40 weeks between 2 Oct 2020 and 2 July 2021 (recall ventilator data is not available for the provinces).

Target		Hosp.	ICU	Vent.	Cases	Deaths	Hosp.	ICU	Vent.	Cases	Deaths
Horizon		1-week					2-week				
Region	CA	6.0	8.38	9.47	10.21	17.72	14.23	15.75	13.29	19.38	24.51
	AB	8.53	16.39	–	15.67	35.55	18.81	22.14	–	29.61	46.08
	BC	8.27	13.33	–	12.50	40.61	16.81	21.65	–	26.51	54.00
	MB	14.68	23.33	–	19.07	39.15	24.88	28.66	–	34.31	48.73
	ON	29.35	9.45	–	12.30	22.77	19.05	16.93	–	20.55	28.60
	QC	8.92	13.89	–	14.22	21.81	18.09	18.80	–	22.43	27.79
	SK	14.89	27.57	–	18.90	38.14	26.56	30.00	–	23.82	49.21

Horizon		3-week					4-week				
Region	CA	22.55	20.07	27.48	29.98	34.67	32.42	27.26	33.26	37.72	56.53
	AB	24.68	34.79	–	42.69	61.77	37.13	45.80	–	52.62	97.42
	BC	25.13	33.18	–	31.40	67.39	29.85	28.70	–	37.72	75.64
	MB	31.62	46.23	–	37.78	77.68	52.28	50.36	–	66.11	100.81
	ON	29.97	22.88	–	32.13	42.12	38.20	28.76	–	27.14	51.88
	QC	24.39	37.89	–	35.66	45.76	33.60	35.04	–	42.95	84.08
	SK	30.23	35.65	–	39.33	58.05	35.33	45.23	–	56.84	79.19

### TCN’s forecasts based on various factors

**Figure 5:**
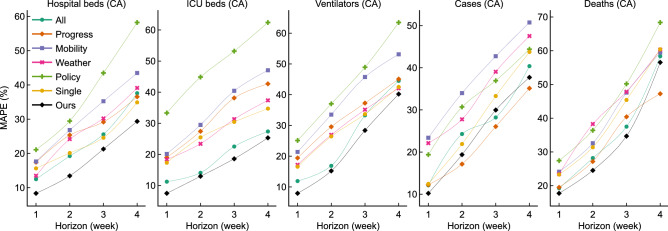
Comparison of the performance (in terms of MAPE) of TCN models using the various factors as input.

Figure 5 shows the performance of TCN models using various sets of factors: all the 27 factors (‘All’), the 3 pandemic progress factors (‘Progress’), the 5 population mobility factors (‘Mobility’), the 5 weather condition factors (‘Weather’), the 12 public policy factors (‘Policy’), and a target-same factor (‘Single’—i.e., the single target factor same, e.g., use only the factor previous ‘number of ICU beds’ to forecast ‘the number of ICU beds’), for forecasting the 5 targets (i.e., number of hospital beds, ICU beds, ventilators, cases, and deaths) in Canada. (Note all the forecasts are made based on the ’history size’ shown in Table 4, i.e., using the history of 2 weeks when forecasting 1 or 2 weeks in the future, and a history of 3 weeks for forecasting 3 and 4 weeks in the future). For all the three hospital resources, our models yield the best forecasts: the MAPE for every 1-week forecast (resp., 2-week, 3-week, 4-week) is below 10% (resp., below 15%, around 20% for hospital and ICU beds and 28% for ventilators, and around 29%, 24%, and 40%, respectively). For the number of cases and deaths, using either the task-specific factors or the 2 progress factors yield the most accurate forecasts. Next, we explored whether the history of data we used was appropriate.

### TCN’s forecasts based on various past values

**Figure 6:**
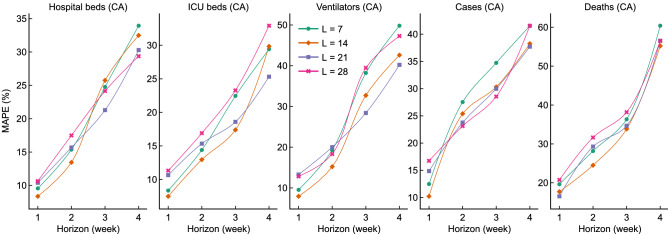
Comparison of the performance (in terms of MAPE) of TCN models using various past values as input.

Figure 6 shows TCN’s forecasts based on various historical data (i.e., the values in the past 1, 2, 3, 4 weeks) of our current specific factors. We see that our choice—i.e., using the values in the past $L=14$ days for 1- and 2-week forecasts and past $L=21$ days for 3- and 4-week forecasts yields relatively lower MAPEs for most tasks: ‘14 days’ (orange) is the best for 4 of the 5 1-week forecasts (except for Deaths), and for 4 of the 5 2-week forecasts (except for Cases). Moreover, the ‘21 days’ (violet) is the best for 3 of the 5 3-week forecasts (except for ICU beds and Cases), and for 3 of the 5 4-week forecasts (except for Hospital beds and Deaths). Note that no other single-history does better.

### Comparisons between TCN and other models

**Figure 7:**
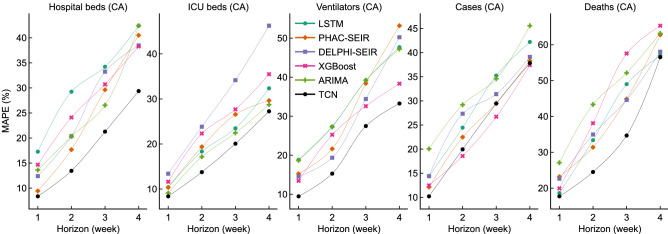
Models’ performance, in terms of MAPE (%) of the 1-,2-,3-,4-week forecasts in *Canada*.

Figure 7 shows the MAPE of the forecasts made by TCN models and the other six competing models between 2 Oct 2020 and 2 July 2021 in Canada. Our models consistently outperform other models for all 12 resource utilization forecasting tasks: TCN’s forecasts are the best, much ahead of the second-best forecasts for the four tasks about hospital beds and the four tasks about ventilators, and slightly ahead of the second-best forecasts (yielded by ARIMA) for ICU-beds. TCN achieves as low as around 28%, 26%, and 33% MAPE for the 4-week forecast of the number of hospital beds, ICU beds, and ventilator forecasts, respectively. Compared to LSTM, TCN achieves an average of 10% MAPE decrease (across the four horizons) for forecasting the number of hospital beds, 4% decrease for ICU beds, and 5% decrease for ventilators. For the four case forecasting tasks, TCN performs best for the 1-week forecast and second-best (XGBoost is the winner) for 2-, 3-, and 4-week forecasts. For the 2- and 3-week forecast of the number of deaths, TCN achieves a 23% and 34% MAPE, respectively, which are much lower than other models.

### Comparisons between TCN and IHME

**Figure 8:**
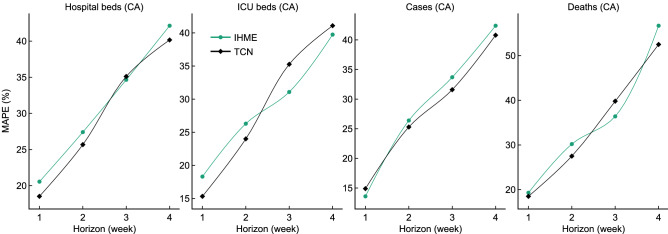
Models’ performance on IHME data, in terms of MAPE (%) of the forecasts in **Canada**.

Figure 8 shows the comparison between TCN’s forecasts and IHME forecasts (which have been released by the IHME team). IHME package has not yet been configured for use outside the IHME infrastructure^14^ and therefore we extended the TCN models to IHME data (*n.b.*: this means that IHME’s “ground truth” is different from ours) and compared the TCN’s forecasts with IHME’s forecasts. We replaced our ground truth (i.e., the number of hospital beds, ICU beds, cases, and deaths) with IHME data and made forecasts. For the four forecasting tasks about hospital beds, TCN yields a much lower MAPE than IHME but the 3-week forecast. For the ICU beds, TCN performs better on the 1- and 2-week tasks and IHME wins the 3- and 4-week tasks. Compared to IHME’s forecasts, TCN’s 2-,3-,4-week forecasts of the number of cases and 1-,2-,4-week forecasts of the number of deaths are more accurate.

### Forecasts during the fourth wave

**Figure 9:**
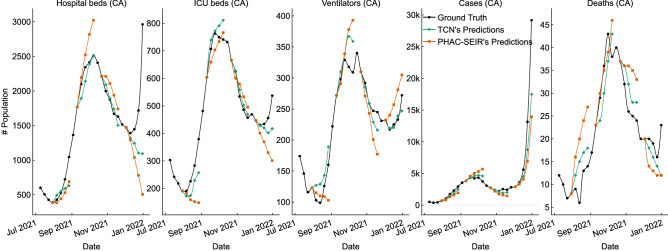
Forecasts made by TCN and PHAC-SEIR on 30 July, 10 Sept, 22 Oct, and 3 Dec 2021 in *Canada*.

We also made forecasts of the five targets during the fourth wave of the COVID-19 pandemic in Canada. Each sub-figure of Fig. 9 gives the forecasts (shown as green and orange dots) made by two models—i.e., TCN (green) and PHAC-SEIR (orange)—on the four dates (i.e., 30 July, 10 Sept, 22 Oct, and 3 Dec 2021—i.e., one forecast each 6 weeks, that span the whole fourth pandemic wave). The forecasts for the 4 time horizons (i.e., the week 1, 2, 3, 4 of the forecasting time point, see the last 4 dots in green (TCN) and in orange (PHAC-SEIR)). Apparently, TCN yields the forecasts (green line) much closer to the ground truth (black line), in comparison to PHAC-SEIR’s forecasts (orange line).

## Discussion

In this work, we develop a TCN-based method to make 116 target-region-horizon-specific forecasts for each of the 40 weeks between 2 Oct 2020 and 2 July 2021: This is one model for each of five Covid-related targets—i.e., the number of hospital beds, ICU beds, ventilators, cases, and deaths—in each of 7 regions (Canada and in each of the six major provinces), and for each of 4 horizons (i.e., the next 1, 2, 3, and 4 weeks).

In the learning process, at each time, the TCN-learner uses just the currently known data (before the forecasting start date)—which are transformed into (*input*,*output*) pairs, then used to produce a learned TCN model, which is then used to produce a specific forecast. Recall the ICU-ON-4 forecast shown in Fig. 1, on 20 Nov 2020, used the known data as of 20 Nov to train the ICU-ON-4(20 Nov 2020) model and the data thereafter to make forecasts.

The results, in Table 5, show that TCN’s forecasts are more accurate than other models: our forecasts for any region, for any target, are closer to the true values, throughout the forecasting period. This is mainly due to TCN’s particular network structure that can explore predictive patterns—i.e., the relationship between input (i.e., the factor values in the past 2 or 3 weeks) and output (i.e., the target values in the next 1, 2, 3, and 4 weeks). Table 5 shows that the MAPE for 4-week forecasts is higher than for the three short-horizon (i.e., 1-,2-,3-week) forecasts, for all tasks; this demonstrates that forecasting for the far future is more challenging than for the near future.

In addition to the forecasts between 2 Oct 2020 and 2 July 2021, we also trained models to forecast for the fourth wave of COVID-19 pandemic in Canada. Figure 9 shows that our models are more accurate (with lower MAPEs) than PHAC-SEIR. More importantly, our models are able to discover the turning point of the pandemic trends. For example, at the end of July when the hospital resource utilizations were decreasing, TCN models successfully forecasted the increase of utilization of ICU beds and ventilators 4 weeks in advance. This indicates that TCN can effectively offer health providers accurate forecasts of resource utilization in advance and therefore allow them to mobilize the hospital resources early, well before the possible surge in demands.

As mentioned earlier, each of our various target-region-horizon-specific models used its own set of factors, over a specified history—e.g., the input factors (as well as the size of their past values) for the Hospital-AB-1 forecast are different from the ICU-ON-4 forecast (i.e., the number of hospital and ICU beds, snowfall, etc., in Ontario; see Fig. 1). To determine whether these features (based on our background knowledge) lead to effective performance, we observed how the performance changed when we learned TCN models based on different factors. As there are 27 factors, each over as many as 21 previous days, there are potentially $2^{27\times 21}$ possible combinations of factors to consider—enough that we are almost guaranteed to overfit if we explicitly considered them all. Instead, we decided to fix the “history” (to either 14 or 21 days, depending on the look-ahead), only use the same factors for all previous times, and only consider the 6 other combinations of the 27 factors (in addition to our current set of task-specific factors, shown in Table 3). The comparison between the current models, using our input features, and the models with other various input factors, demonstrate that our input decisions are effective. The models perform worst for all the hospital utilization forecasting tasks when only using the 12 policy factors as input, and yield high MAPEs for all the pandemic progress forecasting tasks; this suggests a weak relation between the policies and the resource utilization. The forecasts based on only the 5 weather factors are more accurate than on the 5 mobility factors for the hospital beds, ICU beds, ventilators, and cases. When we use only the 2 pandemic progress factors as input, the TCN models can yield more accurate forecasts of the number of cases and deaths, but not the three hospital resources. TCN models using only a single factor (same to the target) can yield relatively accurate forecasts for all the five targets—e.g., the second best 3- and 4-week forecasts for hospital and ICU beds. Our models also performed effectively if using all the 27 factors, although not as good as using our task-specific factors.

We analyzed all the data in the past two weeks (i.e., $L=14$) for 1- and 2-week forecasts and the past 3 weeks (i.e., $L=21$) for 3- and 4-week forecasts. To investigate the effectiveness of this ’history’ setting, we compared our models to new models, that used different history; see Fig. 6. Again, we found that our setting was effective. The four 1-week forecasts (i.e., Hospital-CA-1, ICU-CA-1, Ventilators-CA-1, and Cases-CA-1) and the four 2-week forecasts (i.e., Hospital-CA-2, ICU-CA-2, Ventilators-CA-2, Cases-CA-2, and Deaths-CA-2) are most accurate when $L=14$. When $L=21$, six of the total ten 3- and 4-week forecasts are the best—e.g., the MAPE of ICU-CA-3 and ICU-CA-4 forecasts becomes lower if using $L=21$ in place of $L=28$. In fact, using additional past factor values increases the computation cost but may not further improve the model’s predictive ability—in fact, the $L=28$ setting had the highest MAPE for all the five 1-week forecasts and four 2-week forecasts. Overall, our results show that the data in the past 3 weeks can produce models of pandemic and hospital resource utilization that can accurately forecast for the following 3 weeks.

### Limitations

Although our task-specific models produced good forecasting results, they were based on assumptions that are hard to explain. One of our future directions is to develop an automated method to automatically learn the best subset of factors for each forecast. Our analyses considered the 27 factors shown in Table 3; as this was focused on the first three waves, when the 2-dose vaccination rate in Canada is still very low and few variants had been reported, we did not consider vaccination rate and virus variants. We anticipate these, and perhaps other COVID-19-related factors, will further improve the forecast accuracy. Third, we will explore the latent relationship between the factors and the development of the pandemic and the utilization of hospital resources, hoping to produce a model that could ask important questions like, “How many more/fewer ICU beds would Ontario need for the COVID-19 patients if we change a public policy (e.g., school closing)?” We also recognized that the long-horizon forecasts are crucial to healthcare providers for mobilizing the necessary resources in advance, meaning it would be more useful to make forecasts yet further in the future—e.g., for 5 weeks or longer. Another interesting direction is exploring the demand for hospital resources based on our forecasts; note this will require real-time data about the current hospital capacity across the local hospitals in each region. Forecasting the demand for the resources poses a great challenge, which differs from the resource utilization forecast—e.g., overestimating this demand may lead to severe wasting of resources for local hospitals that are able to deal with COVID-19 patients, and may then lead to resource shortages for other non-COVID hospitals.

## Conclusions

This paper provided a method that learns TCN-models that can forecast many useful healthcare quantities, including the need for hospital beds, ICU beds, and ventilators. These models incorporate the complex interplay of many factors—including regional mobility, weather, and public policy—to produce accurate forecasts that can help decision-makers make effective, accurate decisions. We have provided a method for effectively learning these TCN models for various target-region, and horizon-specific forecasts, including the weekly average number of hospital beds, ICU beds, ventilators, cases, and deaths in Canada and six provinces (AB, BC, MB, ON, QC, SK) for up-to-4 weeks in the future. The numerous experiments demonstrated that our method is more accurate (in terms of MAPE) than four state-of-the-art predictive models. We also demonstrated that our method can accurately forecast the weekly average number of cases and deaths in the future.

## Supplementary Information

Supplementary Figures.

## Data Availability

All the data used in our analyses are available online, where the links have been presented in the paper.
