# Supplementary material for: Learning models for forecasting hospital resource utilization for COVID-19 patients in Canada
Source: Sci Rep. 2022 May 24;12:8751. doi: 10.1038/s41598-022-12491-z (PMC9128327; doi:10.1038/s41598-022-12491-z)
Supplement: Supplementary file 1 — Supplementary Figures. [file 41598_2022_12491_MOESM1_ESM.pdf]

Learning Models for Forecasting Hospital Resource Utilization for COVID-19 Patients in Canada

Supplemental Figures

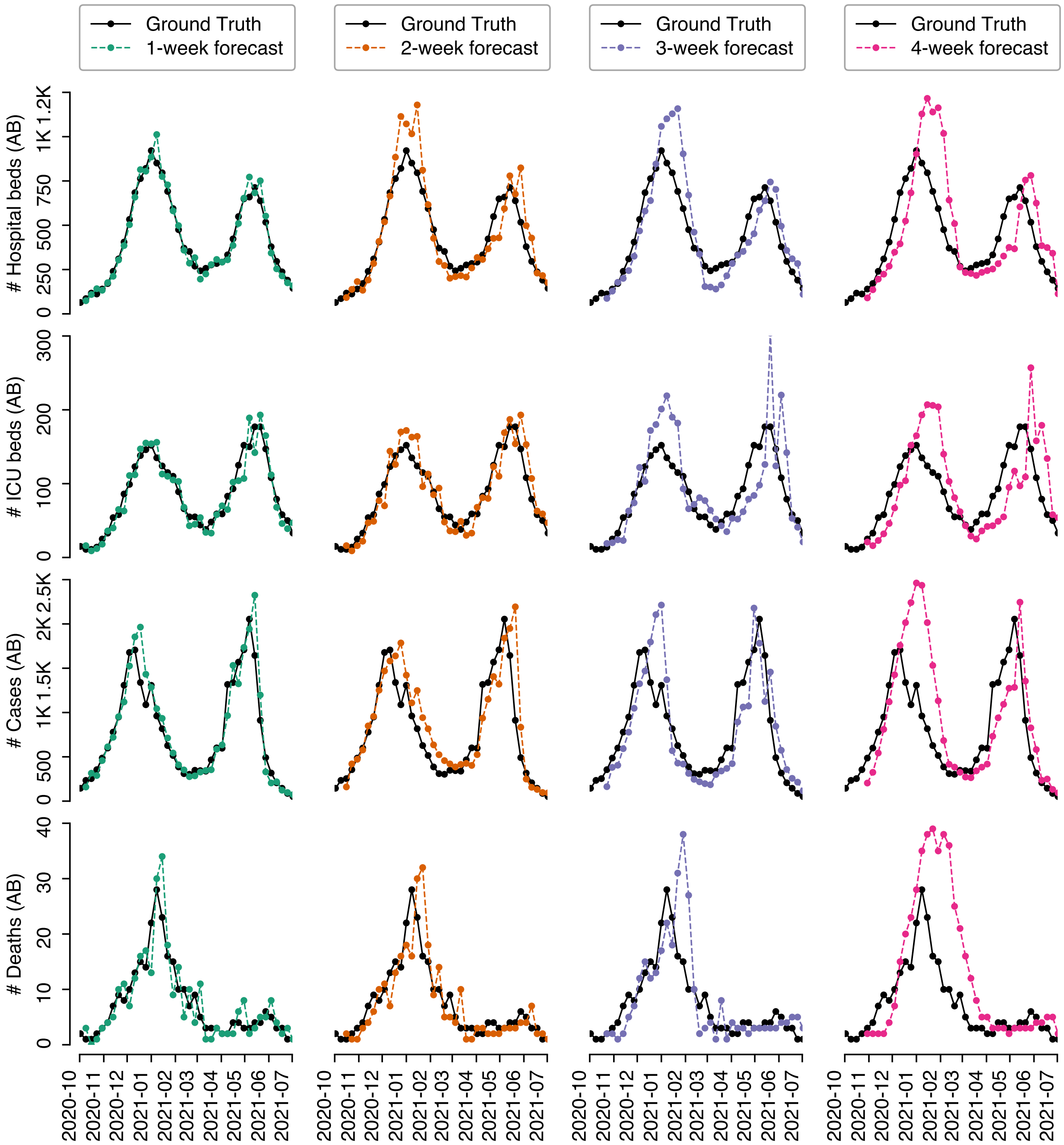

Supplemental Figure S1. 1-,2-,3-,4-week forecasts in **Alberta**.

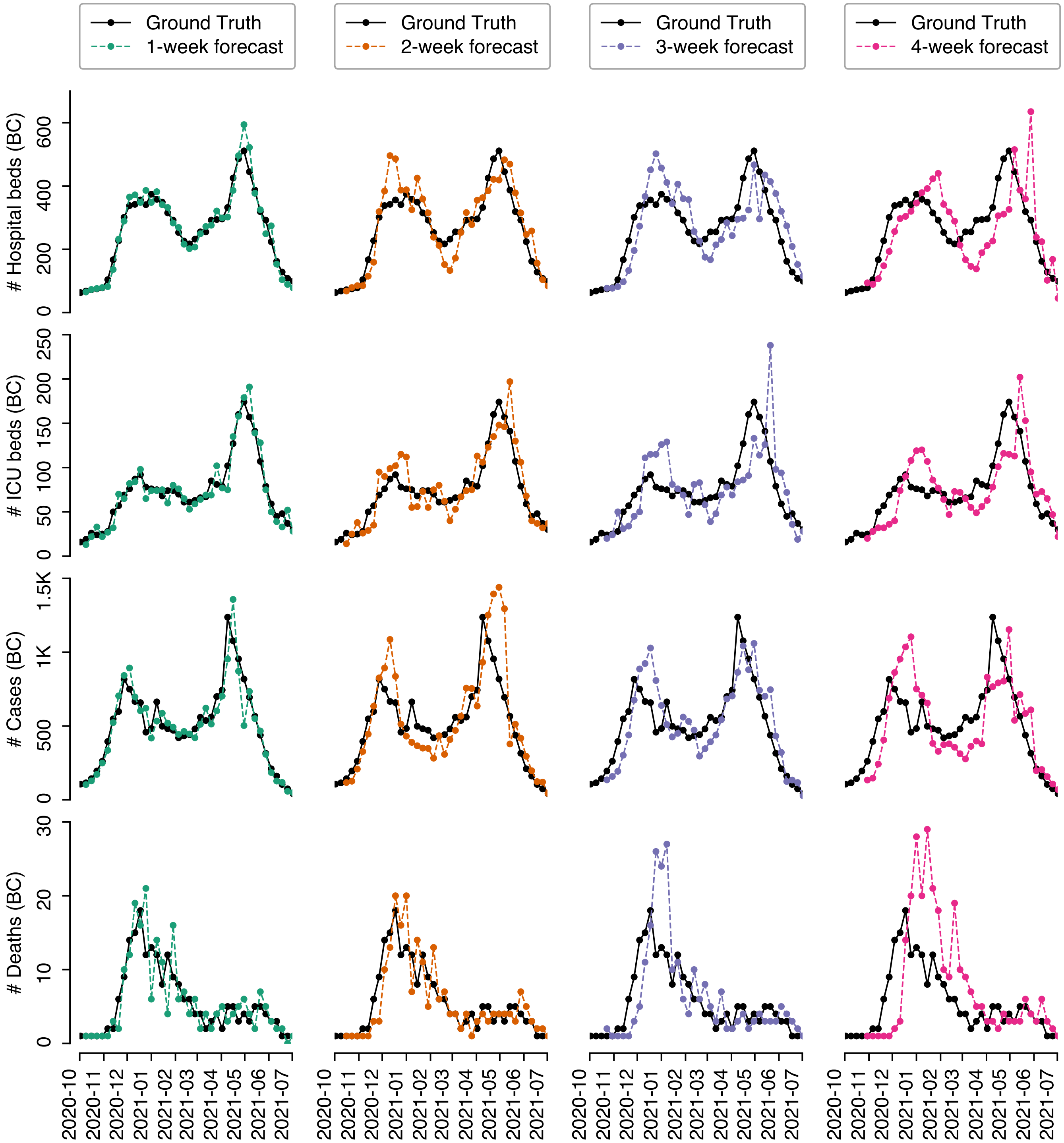

Supplemental Figure S2. 1-,2-,3-,4-week forecasts in **British Columbia**.

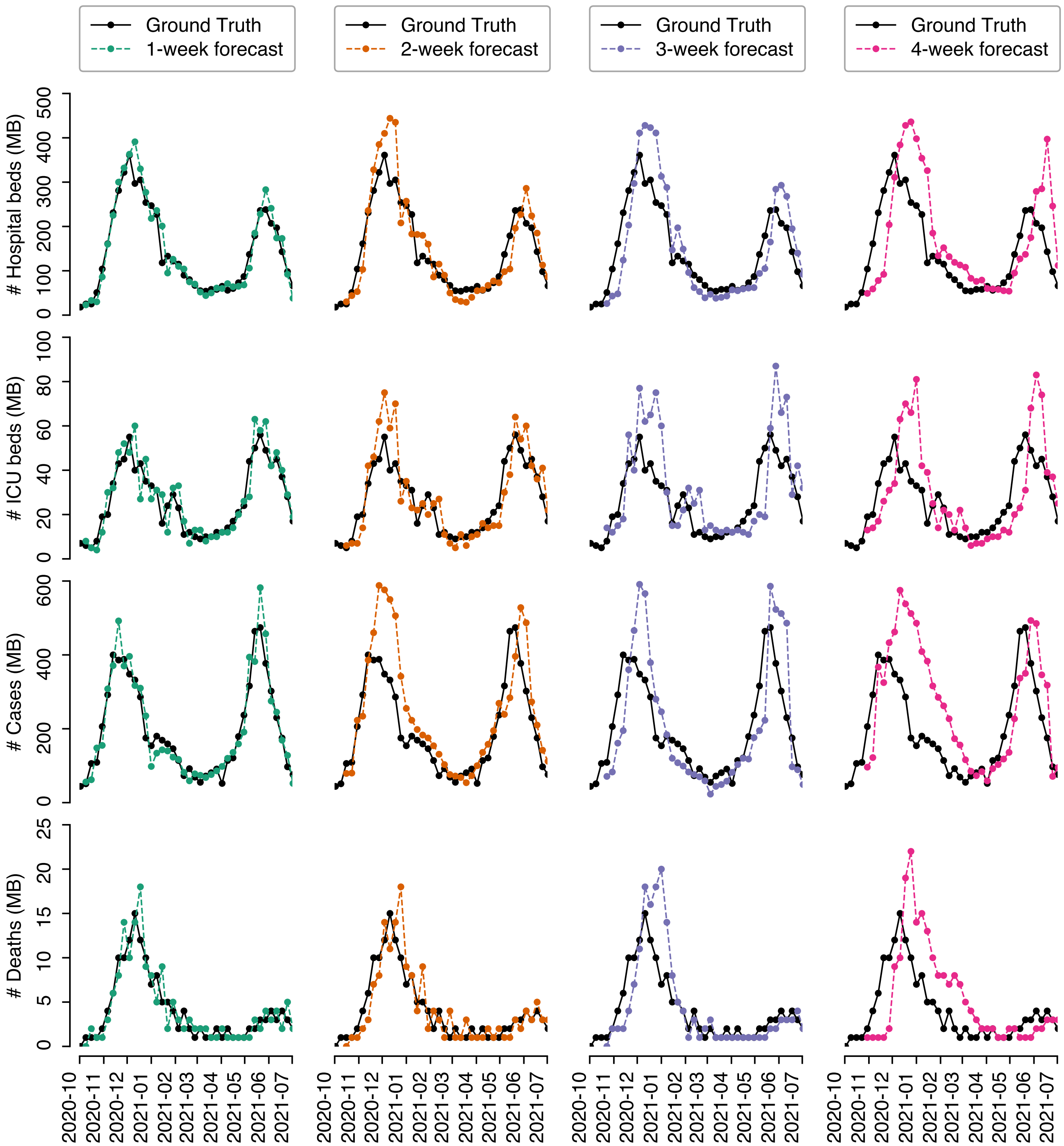

Supplemental Figure S3. 1-,2-,3-,4-week forecasts in **Manitoba**.

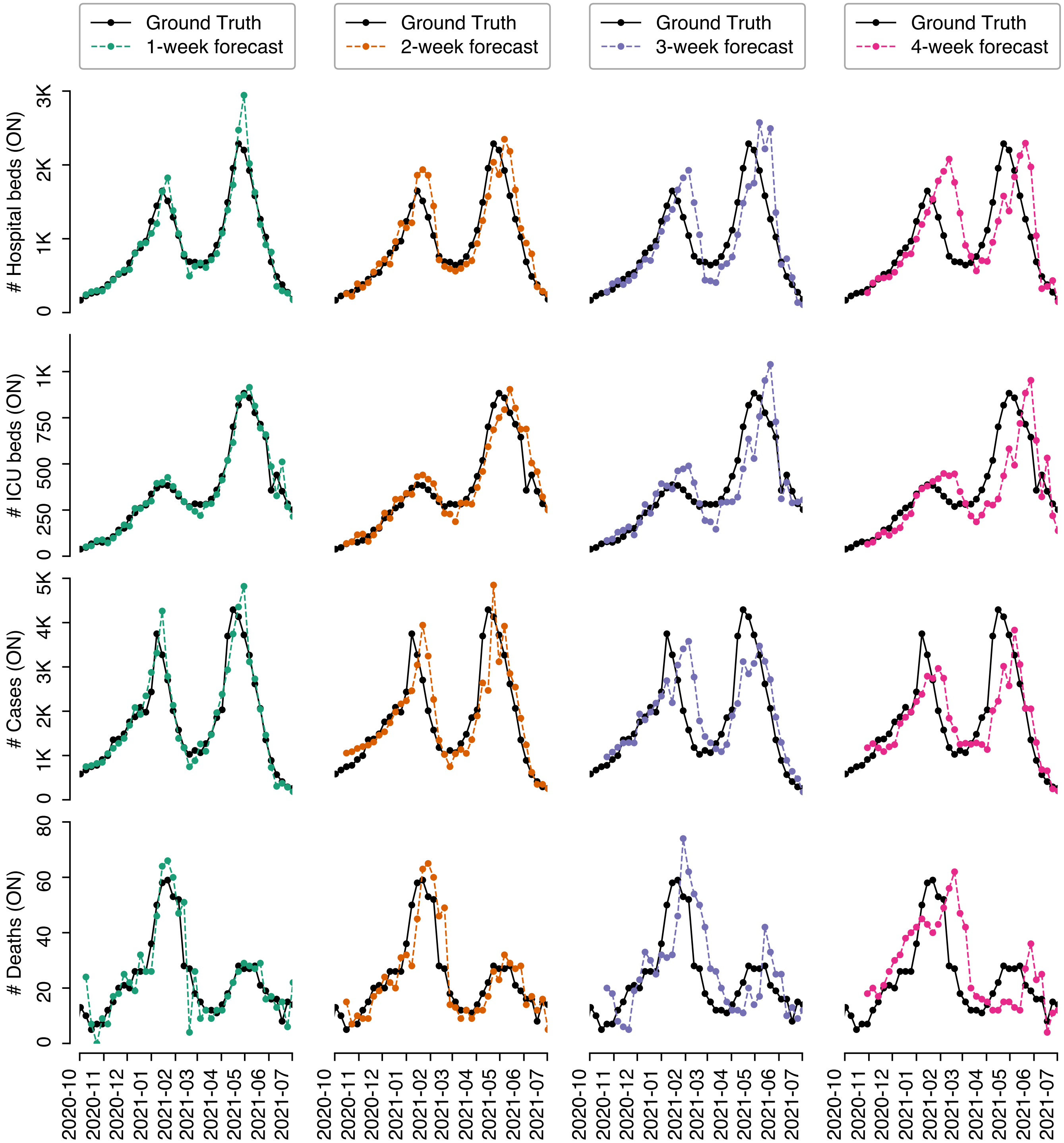

Supplemental Figure S4. 1-,2-,3-,4-week forecasts in **Ontario**.

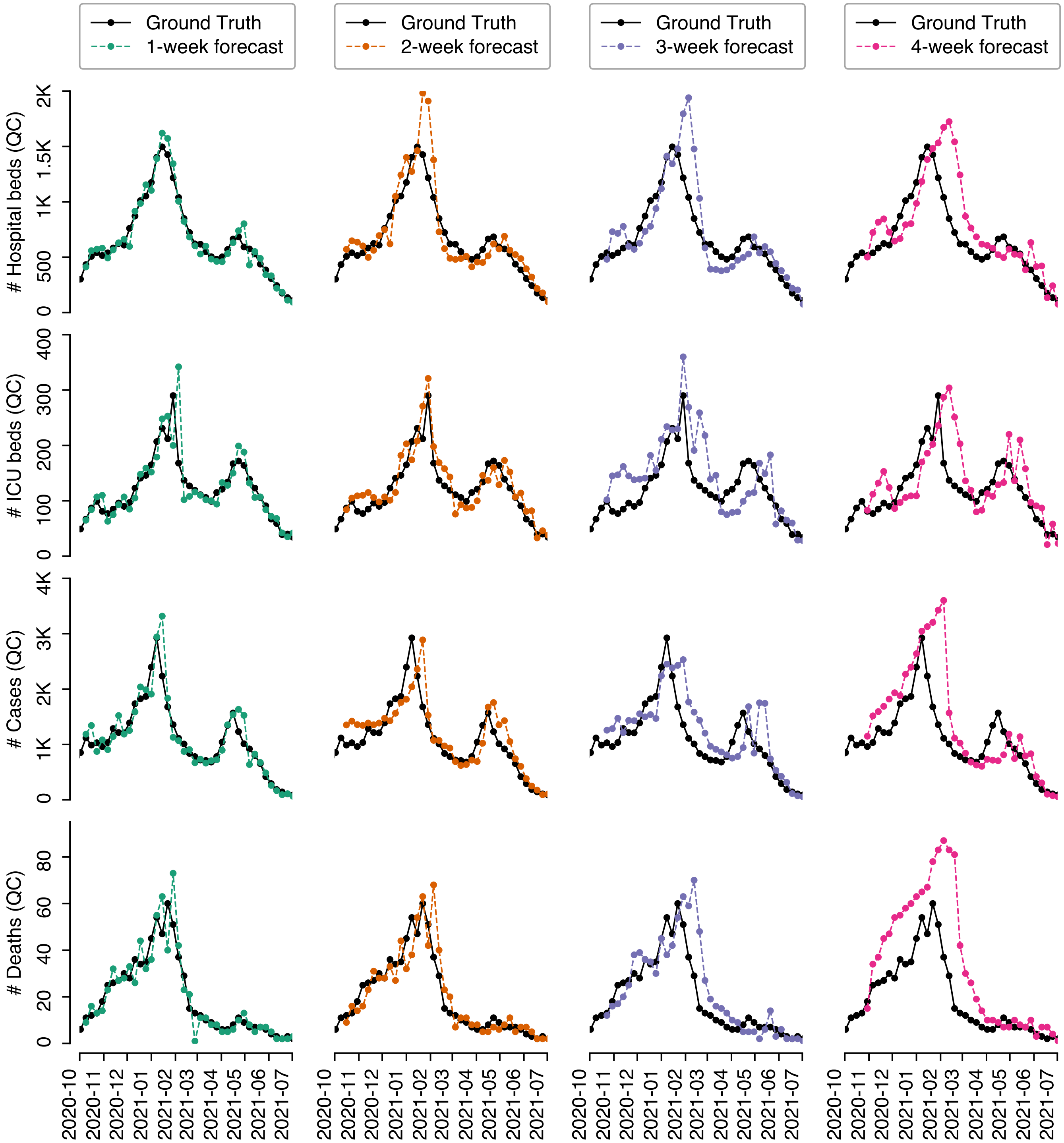

Supplemental Figure S5. 1-,2-,3-,4-week forecasts in **Québec**.

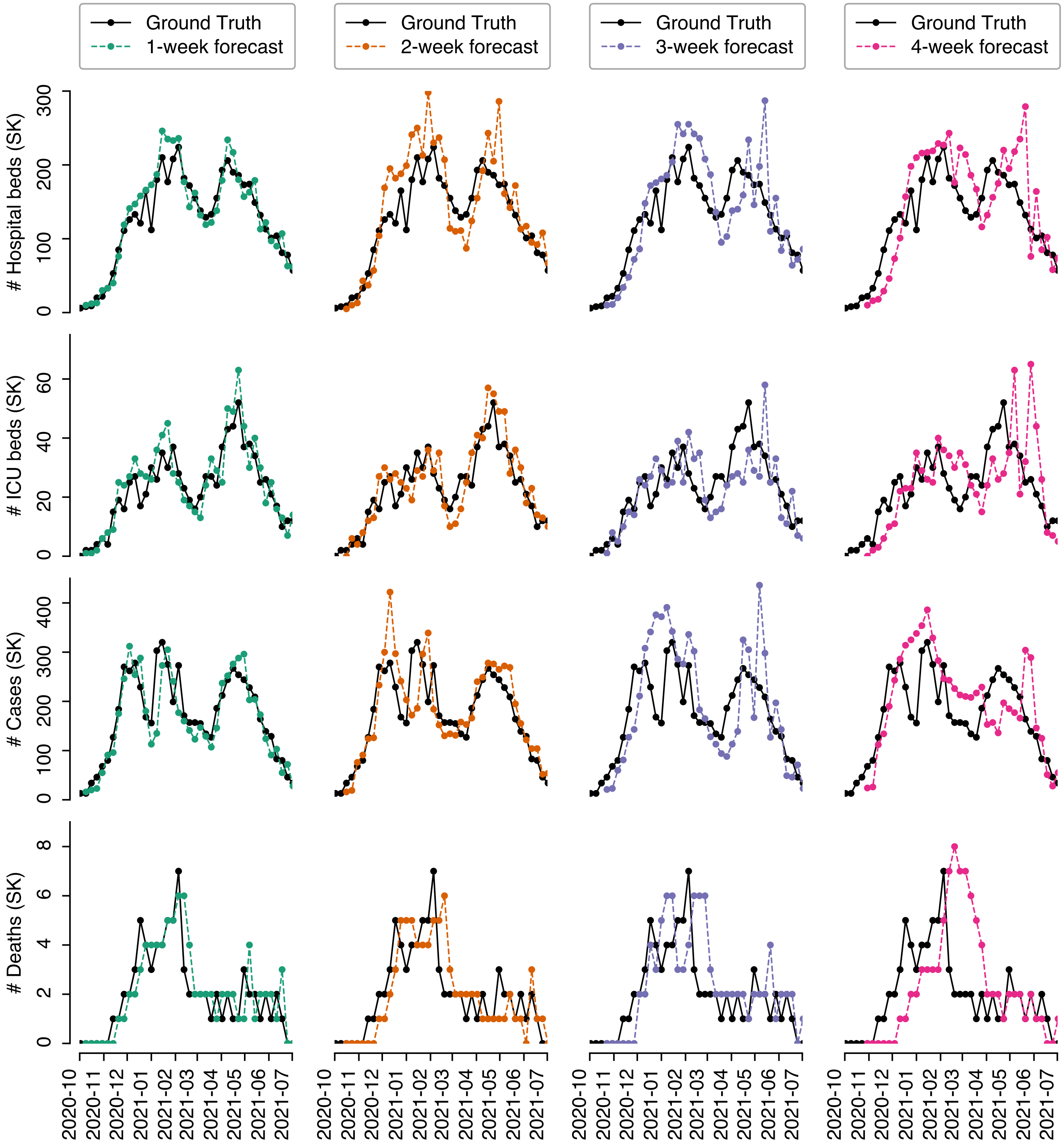

Supplemental Figure S6. 1-,2-,3-,4-week forecasts in **Saskatchewan**.
